# The mitochondrial L-lactate dehydrogenase affair

**DOI:** 10.3389/fnins.2014.00407

**Published:** 2014-12-09

**Authors:** Salvatore Passarella, Gianluca Paventi, Roberto Pizzuto

**Affiliations:** Department of Medicine and Health Sciences, University of MoliseCampobasso, Italy

**Keywords:** mitochondria, L-lactate dehydrogenase, L-lactate metabolism, gastrocnemious muscle mitochondria, energy metabolism

The existence of a mitochondrial L-lactate dehydrogenase (m-L-LDH) suggested by Dianzani (1951), was shown by Baba and Sharma (1971) with the enzyme located in the mitochondrial matrix; later Brooks et al. (1999) proposed the intracellular lactate shuttle and in the third millennium the existence of m-L-LDH was definitively been confirmed in mammalian, plant and yeast mitochondria as reviewed by Schurr (2006), Passarella et al. (2008), and Brooks (2009), being its existence finally recognized by inclusion of m-L-LDH in the Mitocarta (http://www.broadinstitute.org/pubs/MitoCarta/index.html). The experimental strategy to be used to show whether and how L-lactate can enter mitochondria to be metabolized is well-established and has been applied to a variety of mitochondria including heart (Brooks et al., 1999; Valenti et al., 2002), liver (Brooks et al., 1999; de Bari et al., 2004), skeletal muscle (Dubouchaud et al., 2000; de Bari et al., 2008; Passarella et al., 2008) plant (Paventi et al., 2007), brain (Schurr, 2006; Atlante et al., 2007; Schurr and Payne, 2007; Hashimoto et al., 2008), and cancer cells (de Bari et al., 2010a; Pizzuto et al., 2012). Thus, it is a matter for considerable surprise that the overwhelming evidence for an m-L-LDH located inside mitochondria is not by now universally accepted (Rasmussen et al., 2002; Sahlin et al., 2002; Ponsot et al., 2005; Gladden, 2007; Yoshida et al., 2007; Elustondo et al., 2013).

Using correctly applied procedures, metabolism of L-lactate via the m-L-LDH can be investigated in mitochondria (but also in permeabilized cells, cell homogenates, and mitoplasts) in a few hours. Obviously, caution must be used to control that mitochondrial coupling was not impaired. This simple strategy includes both L-lactate uptake measurements and measurements of mitochondrial processes occurring after L-lactate uptake due to the occurrence of an enzyme i.e., m-L-LDH, which can metabolize the imported L-lactate with in some cases export of the newly synthesized metabolites (for some details see Passarella et al., 2003). The measurements include:

1. Swelling measurements which provide initial evidence that L-lactate can enter mitochondria; importantly, the stereospecificity of the process and the inhibition of swelling found due to non-penetrant compounds strongly suggest that L-lactate uptake occurs in a carrier-mediated manner. Obviously a carrier-mediated transport itself suggests that L-lactate is metabolized inside mitochondria.
2. Measurements of the increase in the redox state of the intramitochondrial pyridine nucleotides found as a result of the addition of L-lactate to the mitochondrial samples; such an increase itself shows that mitochondrial metabolism occurs inside the organelles via the NAD^+^ dependent m-L-LDH. Having established, by applying the control strength criterion (see Passarella et al., 2003), that the rate of NAD^+^ reduction mirrors that of L-lactate transport across the mitochondrial membrane, the transport kinetics can be investigated including their pH and temperature dependence. Importantly the presence of carrier/s devoted to transport L-lactate across the mitochondrial membrane postulates that m-L-LDH is located inside mitochondria. Changes in NAD^+^/NADH redox state should be modulated by certain ionophores under conditions designed to selectively affect Δ pH and Δ Ψ as well as inhibited by a variety of non-penetrant compounds. In the former case the nature of the energy dependence of the transport can be established; in the latter the inhibition profiles could be used to ascertain further whether the L-lactate carriers, the pyruvate carrier and other carriers differ from one another. In some cases this has been shown: in distinction with others we have shown that two separate carriers transport pyruvate and L-lactate into rat liver mitochondria (de Bari et al., 2004). The point is that α-cyano-hydroxy-cinnamate (α-CCN^−^) can inhibit the uptake of both pyruvate and L-lactate, but the pyruvate carrier is inhibited at a concentration (25 μM) at which no inhibition of L-lactate transport occurs.
3. Measurements of oxygen consumption by coupled purified mitochondria due to L-lactate addition. To conclude that oxygen consumption depends on the NAD^+^ dependent m-L-LDH inhibition by the complex I inhibitor rotenone as well as by oxalate/oxamate, inhibitors of L-LDH must be found.
4. Proton efflux and increase of membrane potential could be also found as a result of L-lactate uptake and metabolism. Conversely proton uptake occurs as a result of L-lactate addition to mitochondria previously treated with an inhibitor cocktail used to prevent any energy metabolism. These effects show the existence of L-lactate energy metabolism via m-L-LDH and of a proton compensated L-lactate symport.
5. Measurements can be made to show the efflux of a variety of metabolites newly synthesized inside mitochondria due to externally added L-lactate. This could occur via antiporters, separate from the L-lactate, D-lactate, and pyruvate carriers. The *in vitro* reconstruction of the L-lactate/pyruvate shuttle and of gluconeogenesis (Valenti et al., 2002; de Bari et al., 2004) has been shown and suggests that the mitochondrial L-lactate metabolism is associated with gluconeogenesis occurring perhaps together with L-lactate oxidation to pyruvate in the cytosol. However, even efflux of certain metabolites via unidentified carrier/s show the occurrence of intramitochondrial L-lactate metabolism (Pizzuto et al., 2012).

Use could be made of arsenite, inhibitor of the pyruvate dehydrogenase, to rule out that oxygen consumption, proton efflux, Δ Ψ increase and metabolite traffic derives solely from the metabolism of pyruvate generated from L-lactate in external mitochondrial compartments and taken up in the matrix.

Last, but not least, (a) enzymatic assay (b) immunological analysis, and (c) confocal fluorescence microscopy can be used: (a) allows for initial information about enzyme kinetics features; (b) and (c) can reveal the existence of m-L-LDH even if inactive, but have the limitation that they are of no value in dissecting L-lactate metabolism.

A part of this strategy has unsuccessfully been applied in other laboratories with the conclusion that m-L-LDH either does not exist or is localized in the outer mitochondrial compartments. This has led to the mistaken conclusion that L-lactate is not a mitochondrial metabolite despite all the evidence to the contrary. In this case skeletal muscle mitochondria were investigated. Our opinion is that perhaps the investigators concerned were not able to isolate coupled mitochondria, a task that is far from easy in particular with skeletal muscle samples, and that they were not careful enough in selecting reaction media and in using inhibitors at the right concentrations. For instance the failure to measure oxygen consumption as a result of L-lactate addition to skeletal muscle mitochondria (Elustondo et al., 2013) could be due to the presence of 5 mM MgSO_4_in the medium used to prepare isolated skeletal muscle mitochondria: sulfate is known to enter mitochondria causing efflux of intramitochondrial phosphate, malate, and succinate (Crompton et al., 1974, 1975). Moreover, 60 mM lactobionate included in the medium used to measure oxygen uptake by mitochondria is expected to prevent L-lactate uptake due to its chemical structure and high concentration. Finally, no control has been reported that c and m-L-LDH dehydrogenase are insensitive to 60 mM lactobionate. Of course, it is impossible to mimic cytosol totally with the medium used for *in vitro* experiments, however if findings observed in medium free of compounds expected to affect the mitochondria, “disappear” in another medium a detailed examination of the experimental conditions is needed.

Our opinion is that, having established that mitochondria are purified and coupled, a simple protocol, used in the experiment show here (Figure 1), if properly followed will definitively ascertain the existence of m-L-LDH as well as its localization.

**Figure 1:**
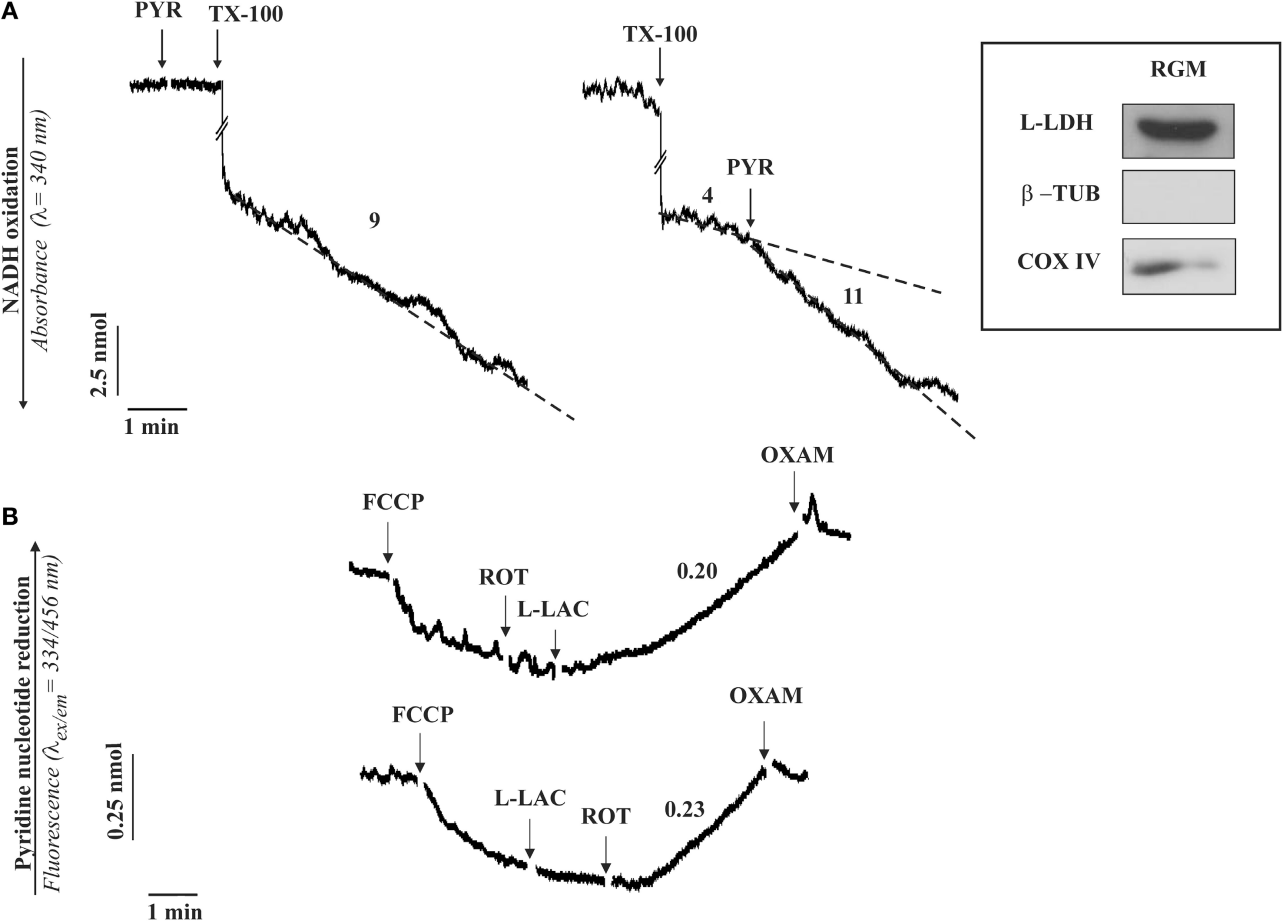
**The occurrence of a L-LDH (A) and L-lactate metabolism (B) in isolated rabbit gastrocnemius mitochondria (RGM). (A)** Enzymatic assay of L-LDH in RGM. RGM (0.15 mg protein) were incubated at 25°C in 2 ml standard medium consisting of 0.2 M sucrose, 10 mM KCl, 1.5 mM MgCl_2_, 1 mM Na-EDTA, 20 mM HEPES-TRIS (pH 7.2) in the presence of NADH (0.2 mM) and the absorbance (λ = 340 nm) was continuously monitored. At the time indicated by the arrows the following additions were made: pyruvate (PYR, 1 mM), Triton X-100 (TX-100, 0.1%). Numbers along curves are rates expressed as nmol NAD(P)H oxidized/min x mg mitochondrial protein. **(B)** Fluorimetric investigation of the redox state of the mitochondrial pyridine nucleotides caused by L-lactate (L-LAC) addition to RGM. RGM (0.5 mg protein), were incubated at 25°C in 2 ml standard medium and reduction of pyridine nucleotides was followed fluorimetrically (λ_*ex*_ = 334 nm; λ_*em*_ = 456 nm) as a function of time. At the arrows the following additions were made: FCCP (1 μM), rotenone (2 μg), L-LAC (5 mM), oxamate (OXAM, 5 mM). Numbers along curves are rates expressed as nmol of NAD(P)^+^ reduced/min × mg mitochondrial protein. Inset: immunodetection of L-LDH in RGM. Solubilized protein (35 μg) from mitochondrial fraction was analyzed by Western blotting as described in Pizzuto et al. (2012). Membrane blots were incubated with polyclonal anti-L-LDH, anti-COX-IV and anti-β-tubulin (β-TUB). COX-IV and β-TUB were used as mitochondrial and cytosolic markers, respectively. The experiment shown in this figure has been described in Passarella et al. (2008) and reported in de Bari et al. (2008).

Rabbit gastrocnemius muscle is rapidly isolated (5–10 min) after killing the animals and put immediately in ice-cold KCl medium (0.1 KCl, 50 mM Tris-HCl, 5 mM MgCl_2_, 1 mM EDTA, 1 mM ATP, pH 7.5). Mitochondria (RGM) are isolated as in Lee et al. (1993) with the exclusion of protease treatment and immediately checked for their intactness by showing that no reduction of absorbance at 334 nm occurs as a result of NADH addition. m-L-LDH activity is assayed in RGM solubilized with 0.1% Triton X-100 (TX-100) as the decrease in absorbance of NADH after pyruvate addition (Figure 1A). No absorbance change occurs when pyruvate is added to intact mitochondria, this showing that m-L-LDH is located in the inner mitochondrial compartments. Surprisingly enough, such a simple assay was not reported in a paper in which the m-L-LDH existence was denied since “the distribution of L-LDH activity among the fractions paralleled that of pyruvate kinase” (Rasmussen et al., 2002); notice that the occurrence of a mitochondrial pyruvate kinase was later shown (Pizzuto et al., 2010). In another case the L-LDH activity was considered negligible (Yoshida et al., 2007). Thus, in our opinion it is easy to dismiss the possibility that the m-L-LDH is located in the outer mitochondrial membrane/intermembrane space: no NADH oxidation occurs when pyruvate is added to purified mitochondria, whereas treatment of mitochondria with TX-100 results in NADH oxidation via Complex 1 with rate increased by addition of pyruvate which reacts with NADH via m-L-LDH.

According to (2), the existence of a m-L-LDH localized in the inner mitochondrial compartments can be simply established by checking the ability of externally added L-lactate to reduce the intramitochondrial NAD^+^ (Figure 1B); in this case addition of L-lactate to mitochondria (previously treated with the uncoupler FCCP, which favors the oxidation of the intramitochondrial NADH, and with the complex I inhibitor rotenone to prevent any oxidation of the newly formed NADH) results in an increase of the NAD(P)H fluorescence. The involvement of L-LDH in this process is confirmed by the inhibition due to oxamate, an inhibitor of LDH.

Consistently, immunological analysis shows that in mitochondria free of cytosolic contamination (no tubulin, a marker of the cytosolic fraction) contains a protein recognized by the L-LDH antibody (inset Figure 1).

We wonder why other investigators who deny the existence of mitochondrial L-LDH did not carry out these simple experiments. Perhaps their views are colored by the mistaken belief, based on incorrect thermodynamic arguments, that mitochondria cannot import L-lactate. Indeed both Sahlin et al. (2002) and Rasmussen et al. (2002) argue at the idea of L-lactate conversion to pyruvate inside mitochondria is not feasible on the basis of thermodynamic principles. They point to a much higher reduction of the NAD^+^/NADH redox couple inside mitochondria; so much higher that in fact it would theoretically eliminate the possibility of L-lactate to pyruvate conversion. Sahlin et al. (2002) go on to suggest that if m-L-LDH were present in the mitochondrial matrix, it would lead to a futile cycle in which pyruvate would be reduced to L-lactate in mitochondria and vice versa in the cytosol, oxidizing mitochondrial NADH and finally removing the driving force for the electron transport chain. However, given that in brain mitochondria the NAD^+^ concentration is 8–20 fold higher than that of NADH and that pyruvate is actively oxidized via pyruvate dehydrogenase, we suggested (Atlante et al., 2007) that m-L-LDH *in vivo* catalyzes essentially L-lactate oxidation. Ultimately, the removal of the oxidation product by carrier-mediated transport and mitochondrial metabolism overcomes any thermodynamic difficulty. In this case, our results were consistent with the postulate/proposal of Schurr (2006) that L-lactate is the only major product of cerebral glycolysis and that it can be metabolized inside mitochondria. On the other hand glucose oxidation to L-lactate is expected to occur when oxidative phosphorylation is reduced since citrate and/or other citric cycle intermediates are required outside mitochondria for anabolism to occur (e.g., see Pizzuto et al., 2012). In this case anaerobic glycolysis is expected to provide ATP and L-lactate to play in mitochondria an anaplerotic role and/or to be transferred to other cells in the intercellular shuttle.

Notice that a putative L-lactate oxidase, located in the intermembrane space of rat liver mitochondria has been shown (de Bari et al., 2010b). This enzyme gives H_2_O_2_ and pyruvate and could be candidate to the new role proposed for L-lactate as “lactormone,” i.e., in Brooks' term (Hashimoto and Brooks, 2008) as a cell-signaling molecule that is involved in the adaptive response to exercise.

To gain further insight into the physiological role of mitochondrial L-LDH in intact cells such as neurons and myocytes, experiments in which pyruvate but not L-lactate metabolism is prevented should be carried out, otherwise mitochondria should be used. Likely low α-cyanocinnamate concentrations and arsenite could be used to prevent any pyruvate metabolism. Notice that in Hep G2 cells as in cancer cells, the mitochondrial pyruvate carrier does not work properly (Pizzuto et al., 2012). On the other hand, if pyruvate metabolism cannot be prevented one should be forced to use isolated mitochondria. Indeed, it was claimed that L-lactate uptake by mitochondria and intramitochondrial metabolism was not feasible, due to thermodynamics reasons (see above), but mitochondria did not know this … and took up L-lactate! We are waiting for further progress!

## Conflict of interest statement

The authors declare that the research was conducted in the absence of any commercial or financial relationships that could be construed as a potential conflict of interest.
